# Non-palm Plant Volatile α-Pinene Is Detected by Antenna-Biased Expressed Odorant Receptor 6 in the *Rhynchophorus ferrugineus* (Olivier) (Coleoptera: *Curculionidae*)

**DOI:** 10.3389/fphys.2021.701545

**Published:** 2021-08-09

**Authors:** Tianliang Ji, Zhi Xu, Qingchen Jia, Guirong Wang, Youming Hou

**Affiliations:** ^1^State Key Laboratory of Ecological Pest Control for Fujian and Taiwan Crops, Key Laboratory of Biopesticide and Chemical Biology, Ministry of Education, Fujian Province Key Laboratory of Insect Ecology, College of Plant Protection, Fujian Agriculture and Forestry University, Fuzhou, China; ^2^State Key Laboratory for Biology of Plant Diseases and Insect Pests, Institute of Plant Protection, Chinese Academy of Agricultural Sciences, Beijing, China

**Keywords:** odorant receptor, *Xenopus* oocyte, *Rhynchophorus ferrugineus* (Olivier), α-pinene, tissue expression analysis

## Abstract

The majority of insects rely on a highly complex and precise olfactory system to detect various volatile organic compounds released by host and non-host plants in environments. The odorant receptors (ORs) are considered to play an important role in odor recognition and the molecular basis of ORs, particularly in coleopterans they are relatively poorly understood. The red palm weevil (RPW), *Rhynchophorus ferrugineus* (Olivier) (Coleoptera: *Curculionidae*), is one of the most destructive pests of the global palm industry. Although feeding and egg oviposition behaviors of RPW can be repelled by some non-palm plant volatiles, such as α-pinene, geraniol, or 1-octen-3-ol, there is limited understanding of how RPW recognizes the non-host plant volatiles. In this study, three candidate *RferOrs* were identified from the Rfer-specific clade, and the tissue expression analysis used was mainly expressed in the antennae of both sexes. Functional characterization of *RferOr6*, *RferOr40*, and *RferOr87* was analyzed by using the *Xenopus* oocyte expression system, and the results indicated that *RferOr6*/*RferOrco* was narrowly tuned to α-pinene. The behavioral experiment showed that α-pinene at the concentrations of 10 and 100 μg/μl can cause a significantly repelled behavioral response of RPW. In conclusion, this study reveals that *RferOr6* is an antenna-biased expressed OR used by RPW to detect the volatile compound α-pinene in non-palm plants, and our results provide a foundation for further *in vivo* functional studies of Or6 in RPW, including *in vivo* knockout/knockdown and feeding/ovipositing behavioral studies of RPW and further pest control.

## Introduction

From bacteria to mammals, most living organisms have evolved a powerful olfactory system with remarkable sensitivities and discriminatory ability to detect and discriminate hundreds of chemicals in the environment (Wuichet and Zhulin, 2010; Hansson and Stensmyr, 2011). Series of insect behaviors, such as host-seeking, mating, communication, and avoidance of predators, were all mediated by the olfactory systems (Bentley and Day, 1989; Hansson and Stensmyr, 2011; Gadenne et al., 2016; Fleischer et al., 2018). In insect chemoreception, the task of olfactory signals encoding and the process of chemoelectrical transduction are mainly mediated by three primary families of receptors, including the gustatory receptors, ionotropic receptors, and odorant receptors (ORs) (Suh et al., 2014; Wicher, 2015; Robertson, 2019). Among them, ORs are the largest gene families in chemosensory receptors and expressed in the dendritic membrane of odorant receptor neurons, which is the primary means for insects in the discrimination of different odors (Fleischer et al., 2018). ORs can be divided into odorant receptor co-receptor (Orco) and traditional ORs. Orco is a coessential in ORs functioning and highly conserved between insects (Benton et al., 2006; Brand et al., 2018), while ORs vary significantly between different insects with low sequence homology. Functional characterization of ORs is a key step toward understanding the molecular mechanism of chemical signal recognition in the peripheral olfactory system of insects.

Over the past 20 years, with a rapid progress in the sequencing of genomics and transcriptomics of insect olfactory tissues, a large number of complete or partial repertoires of OR genes have been identified from a variety of species, and the numbers of OR genes vary considerably between species (Montagne et al., 2015). However, it was noted that the functional characterization of ORs has been obviously reported based on *Drosophila melanogaster* (Hallem et al., 2004; Kreher et al., 2005; Hallem and Carlson, 2006; Mathew et al., 2013), *Anopheles gambiae* (Carey et al., 2010; Wang et al., 2010), *Spodoptera littoralis* (de Fouchier et al., 2017), and *Helicoverpa armigera* (Guo et al., 2020). Otherwise, ORs related to pheromones as well as certain non-pheromone odorants have been characterized in a number of lepidopteran species, such as *Bombyx mori* (Sakurai et al., 2004; Nakagawa et al., 2005; Grosse-Wilde et al., 2006), *Heliothis virescens* (Grosse-Wilde et al., 2007; Wang et al., 2011), *Ostrinia nubilalis* (Wanner et al., 2010), and *Cydia pomonella* (Gonzalez et al., 2015; Cattaneo et al., 2017; Wan et al., 2019). In contrast, although the identification of OR gene sequences was greatly facilitated in Coleoptera (Mitchell et al., 2020), the identification of ligands significantly lagged behind, leaving most ORs as orphan receptors.

The red palm weevil (RPW), *Rhynchophorus ferrugineus* (Olivier) (Coleoptera: *Curculionidae*), is arguably one of the deadliest pests for palm trees worldwide (Ju et al., 2011; Wang et al., 2015). RPW is a concealed tissue borer that almost always lives inside the trunk of palm trees, the weevil larva is the major infestation stage which feed on the apical growing point of the trunk of palm trees, and the attack is detected only after the palm has been seriously damaged (Butera et al., 2012; Shi et al., 2014). Since RPW spends most of its life span inside the trunks of palm trees except during copulation and oviposition, it is difficult to find and kill them. Traps loaded with aggregation pheromones (e.g., 4-methyl-5-non-anol and 4-methyl-5-non-anone) and supplemented with palm esters (e.g., ethyl acetate, ethyl propionate, ethyl butyrate, and propyl butyrate) and/or fermenting mixtures of plant tissues have been known as the major environment-friendly methods for controlling RPW (Faleiro, 2006; Vacas et al., 2014). Unfortunately, the attractant power of these pheromones seems to be limited in the field. Consequently, it is urgent to develop effective integrated pest management tactics based on pheromone traps. Guarino et al. (2013) have proposed a new idea of combing use of the pheromones to attract the RPW into the traps with repellent compounds that drive the pest away from the nearby palm trees to prevent subsequent attacks. Previous studies pointed out that some of the EAG-active non-plant volatiles, belonging to the isoprenoids, phenylpropanoid derivatives, and fatty acid derivatives, were added to the trap with pheromones, respectively, and the capture number of RPW was obviously reduced in the field (Guarino et al., 2013, 2015). Therefore, these chemicals are potentially used as repellents of RPW in the field. Non-host plant volatile compounds that repel insects could be a valuable tool for pest management, but there is still limited understanding of how insects systematically discriminate host and non-host plant volatiles. Although 76 OR genes were annotated in the RPW antennal transcriptome (Antony et al., 2016), the current knowledge of the olfactory recognition molecular mechanism of non-host plant volatiles still remains unknown. In this study, three full-length open-reading frame (ORF) candidate *RferOrs* were identified from the seven ORs in the Rfer-specific clade. Then, the qRT-PCR expression analysis was used to understand the expression of genes at different tissues. Last, three candidate *RferOrs* were expressed in the *Xenopus* oocyte system and the two-electrode voltage-clamp (TEVC) technique was used to characterize their functions. Our results would provide the basis not only for the OR function characterization of coleopteran insects but also for the OR functions to combine with the reverse chemical ecology approach to develop higher effective repellents to regulate the behavior of RPW.

## Materials and Methods

### Insect Rearing

The *R. ferrugineus* adults used in this study were obtained from the stock population that was originally collected in 2008 from a Canary Island date palm (Phoenix canariensis Hort. ex Chabaud) on the campus of Fujian Agriculture and Forestry University (FAFU), Fujian, China. Later, the colony of *R. ferrugineus* was established at the growth chambers (PRX-250B-30, Haishu Saifu Experimental Instrument Factory, Ningbo, China) and maintained at 27 ± 1°C, 75 ± 5% of relative humidity with 12 h light and 12 h dark scheduled in the laboratory at the College of Plant Protection. Due to its cannibalistic behavior, each larva was reared individually in a plastic bottle (60 mm in diameter and 30 mm in height; Jiafeng Horticultural Products Co., Ltd., Shanghai, China). The adults for breeding were reared together in plastic bottles (65 mm in diameter and 100 mm in height). To get the ideal humidity and keep the air ventilation, we put a moist paper in the bottom and drilled a 5-mm diameter hole at the neck of each bottle. Small pieces (20 cm × 15 cm × 10 cm) of sugarcane were provided as a food resource for the weevils. The bottles and fresh sugarcane were changed every 2 days. In order to keep high genetic diversity of the laboratory population, new insects were collected seasonally from the field and were mixed with the previous population.

### Chemical Compounds

Twenty-nine chemical compounds were selected for this study including five non-palm plant volatiles, 22 palm plant volatiles, and 2 compounds of aggregation pheromone and purchased from Sigma or J&K Scientific Ltd. (Table 1). In the following electrophysiological recording experiments to investigate the function of OR genes, these stimulated chemical compounds were followed by the stimulus order.

**TABLE 1:** All chemical compounds used for the functional characterization of *RferOr6*, *RferOr40*, and *RferOr87*.

Stimulus compounds	Stimulus order	CAS number	Purity (%)	Company
**Non-host plant volatiles**				
1-octen-3-ol	1	3391-86-4	98	Sigma
citral	2	5392-40-5	98	Sigma
geraniol	3	106-24-1	99	Sigma
Methyl salicylate	4	119-36-8	98	Sigma
α-pinene	5	80-56-8	97	Sigma
**Hosts plant volatiles**				
2-methyl-1-butanol	6	137-32-6	98	Sigma
1-butanol	7	71-36-3	99	Sigma
3-methyl-1-butanol	8	123-51-3	98	Sigma
3-Hexanol	9	623-37-0	97	Sigma
2-Hexanol	10	626-93-7	99	Sigma
Citronellol	11	106-22-9	95	Sigma
2,3-Butanediol	12	513-85-9	98	Sigma
3-Hexanone	13	589-38-8	97	Sigma
2-Hexanone	14	591-78-6	98	Sigma
4-Ethylacetophenone	15	937-30-4	97	Sigma
2-Non-anone	16	821-55-6	98	Sigma
Hexanal	17	66-25-1	96	Sigma
Octanal	18	124-13-0	99	Sigma
Phenylacetaldehyde	19	122-78-1	95	Sigma
4-Ethylbenzaldehyde	20	4748-78-1	97	Sigma
Methyl benzoate	21	93-58-3	98	Sigma
Methyl Salicylate	22	119-36-8	98	Sigma
Butyl benzoate	23	136-60-7	98	Sigma
Ethyl benzoate	24	93-58-3	99	Sigma
Ethyl acetate	25	141-78-6	99	Sigma
Octyl acetate	26	112-14-1	99	Sigma
Butyl butyrate	27	109-21-7	98	Sigma
**Aggregation pheromone of RPW**				
4-Methyl-5-non-anol	28	104170-44-2	99	J&K Scientific Ltd.
4-Methyl-5-non-anone	29	35900-26-6	99	J&K Scientific Ltd.

### RNA Extraction and cDNA Synthesis

The total RNA from ten 7–14-day-old male and female antennae was extracted using Trizol. The cDNA was synthesized by the RevertAid First Strand cDNA Synthesis Kit (Fermentas, Vilnius, Lithuania) for the following experiments.

### Sequence and Phylogenetic Analyses of ORs

Twelve *RferOrs* and *RferOrco* from RPW antennal transcriptome (unpublished data) with seven ORs from three beetle species including *Megacyllene caryae* (Mitchell et al., 2012), *Ips typographus* (Yuvaraj et al., 2021), and *Dendroctonus ponderosae* (Andersson et al., 2013) were used to construct the phylogenetic trees. The gene name and accession number in GenBank are listed in Table 2. The amino acid sequence data were aligned using ClustalX 2.0 software (Thompson et al., 1994). The transmembrane domains (TMDs) of *RferOrs* were analyzed by using TOPCONS^1^. Phylogenetic trees were constructed by using RAxML version 8 with the maximum likelihood method of the Jones–Taylor–Thornton (JTT) model (Tamura et al., 2013).

**TABLE 2:** Genes used to construct the phylogenetic trees are listed with gene names and accession numbers.

Gene name	Accession number	Gene name	Accession number
RferOrco	A0035283	McOr8	–
*RferOr2*	MW979235	McOr10	–
*RferOr6*	MW979236	McOr13	–
*RferOr2*1	MW979237	McOr22	–
*RferOr40*	MW979238	McOr33	–
*RferOr43*	MW979239	McOr37	–
*RferOr47*	MW979240	McOr39	–
*RferOr56*	MW979241	McOr56	–
*RferOr57*	MW979242	ItypOrco	QOI12086
*RferOr60*	MW979243	ItypOr5	GACR01000029
*RferOr66*	MW979244	ItypOr6	GACR01000018
*RferOr67*	MW979245	ItypOr11	GACR01000021
*RferOr85*	MW979246	ItypOr15	GACR01000030
*RferOr87*	MW979247	DponOrco	XP_019768125
McOrco	–	DponOr8	GABX01000004
McOr2	–	DponOr9	GABX01000059
McOr3	–	DponOr82	–
McOr5	–	DponOr83	XP_019766781

*– The accession number has not been deposited in GenBank.*

### Tissue Expression Pattern of *RferOr6*, *RferOr4*0, and *RferOr87*

Real-time quantitative PCR (RT-qPCR) was used to evaluate the expression levels of *RferOr*6, *RferOr40*, and *RferOr87* in different tissues of RPW. Total RNA was isolated from 7- to 14-day-old adults of antennae (A), legs (L), head (H), proboscides (P), and body (B) of both sexes, respectively. Tubulin and β-actin were used as reference genes (Antony et al., 2018). The specific primers were designed using Primer Premier 5.0 (PREMIER Biosoft International, CA, United States) and listed in Supplementary Table 1. The TransStart Tip Green qPCR SuperMix (TransGen Biotech, Beijing, China) was used for RT-qPCR. The RT-qPCR mix (20 μl) contained 1 μl of cDNA template, 0.6 μl of each primer (10 μM), 10 μl of 2 × TransStart Tip Green qPCR SuperMix, 0.4 μl of Passive Reference Dye (50×), and 7.4 μl of nuclease-free water. The RT-qPCR reactions were performed using the Applied Biosystems 7500 Fast. The real-time PCR system (ABI) was performed under the following conditions: 94°C for 30 s, 40 cycles of 94°C for 5 s, and 60°C for 34 s. The melting curve analysis was followed by the fluorescence measurement with one cycle of 95°C for 15 s, 60°C for 1 min, 95°C for 30 s, and 60°C for 15 s and suggested amplification of a single product. The RT-qPCR experiments were repeated three times using three independently isolated RNA samples. The relative expression level of genes was calculated using the 2^–ΔΔ*SM1Ct*^ method (Livak and Schmittgen, 2001). Statistical significance between males and females was analyzed by using Student’s *t*-test, and relative expression levels of antennal expression of three candidate genes were compared by using one-way ANOVA with IBM SPSS Statistics 25.0 (IBM Corp., Armonk, New York).

### Cloning the Full Length of *RferOr6*, *RferOr4*0, and *RferOr87*

According to the tissue expression pattern, we cloned the full-length *RferOr6*, *RferOr40*, *RferOr87*, and *RferOrco* genes of RPW. Primers of ORs were designed using Primer Premier 5.0 and are listed in Supplementary Table 1. The ORF of ORs was amplified by using PCR from antennal cDNA. The PCR reaction was performed in 50 μl solution containing 25 μl of 2 × PrimeSTAR mix, 2 μl each of upstream and downstream primers (10 μM), and 19 μl of ddH_2_O. The reactions were carried out using a Veriti Thermal Cycler (Applied Biosystems, Carlsbad, CA, United States) under the following conditions: 95°C for 3 min, 35 cycles of 95°C for 30 s, 62°C for 30 s, 72°C for 1.5 min, and 72°C for 10 min (*RferOr6* and *RferOr40*); 95°C for 3 min, 35 cycles of 95°C for 30 s, 61°C for 30 s, 72°C for 1.5 min, and 72°C for 10 min (*RferOr87*); 95°C for 3 min, 35 cycles of 95°C for 30 s, 60°C for 30 s, 72°C for 1.5 min, and 72°C for 10 min (*RferOrco*). PCR amplification products were purified with 1.0% agarose gels, ligated into the pEASY-Blunt vector (TransGen Biotech, Beijing, China), and sequenced by BGI (Beijing, China).

### Receptor Expression in *Xenopus* Oocytes and TEVC Recordings

*RferOr6*, *RferOr40*, *RferOr87*, and *RferOrco* were subcloned into eukaryotic expression vector pT7TS using the ClonExpress II One Step Cloning Kit (Vazyme Biotech Co., Ltd., China). The sequences of the specific primer bearing a Kozak consensus are listed in Supplementary Table 1. The constructed pT7TS vectors were linearized by restriction enzymes (*Apa*I and *Not*I) (Supplementary Table 1), and cRNAs were synthesized by using the mMESSAGE mMACHINE T7 Ultra Kit (Ambion, Austin, TX). Mature and healthy *Xenopus* oocytes were separated and then treated for 1 h at room temperature with washing buffer (2 mM KCl, 96 mM NaCl, 5 mM HEPES, and 5 mM MgCl_2_; pH 7.6 adjusted with NaOH) that contained 2 mg/ml of collagenase. Stock solutions (1 M) of all the compounds used for TEVC recordings were prepared in dimethyl sulfoxide (DMSO) and stored at –20°C. Before the experiments, they were diluted in 1 × Ringer’s buffer (2 mM KCl, 96 mM NaCl, 5 mM MgCl_2_, 5 mM HEPES, and 0.8 mM CaCl_2_, pH 7.6) up to a working concentration of 10^–4^ M. Ringer’s buffer (1×) containing 0.1% of DMSO was used as a negative control. For dose–response plots, serial dilutions were made at 10^–6^, 1 × 10^–5^, 10^–4^, 3 × 10^–4^, and 10^–3^ M. Before injection, the cRNA concentration of *RferOrs* and *RferOrco* was diluted to 2 μg/μl, and then, we mixed the candidate *RferOr* with *RferOrco* in the ratio of 1:1. Later, a volume of 27.6 nl of *RferOr*/*RferOrco* mix was microinjected into each mature oocyte. Then, the injected oocytes were cultured for at least 3 days at 18°C in 1 × Ringer’s solution supplemented with 5% of dialyzed horse serum, 50 μg/ml of tetracycline, 100 μg/ml of streptomycin, and 550 μg/ml of sodium pyruvate. Using TEVC (Warner Instruments, Hamden, CT, United States) at a holding potential of −80 mV and amplified by an OC-725C oocyte clamp amplifier, the oocytes were exposed to each chemical for 20 s and washed with 1 × Ringer’s buffer to bring the current back to baseline before the next stimulus. The data acquisition and analysis were performed with Digidata 1440 A and PCLAMP 10.2 software (Axon Instruments Inc., Union City, CA, United States). The dose–response data were analyzed by using GraphPad Prism 5.0 software.

### Behavioral Experiment

Two arms of the Y-tube olfactometer (diameter: 5 cm, main-tube length: 15 cm, arm length: 30 cm, and arm angle: 75°) were used to observe RPW behavioral response to α-pinene. The one arm of the Y-tube olfactometer was connected to the odor source, containing a piece of filter paper with 10 μl of a mix of two components (mix G contains pheromone and α-pinene; ratio of pheromone:α-pinene = 1:1) and the other to the blank, containing a piece of filter paper with 10 μl of pheromone. The air was filtered through activated charcoal, and the entry flow to the olfactometer was 1.2 L/min controlled by using a vacuum pump. RPW was starved overnight, and the experiment was carried out during daytime between 9:00 and 17:00 h at a temperature of 27°C ± 1°C and a humidity of 75 ± 5% (Antony et al., 2021). During the experiment, the Y-tube olfactometer was washed with ethanol and heated every time, and the compound position was changed when every 10 adults were tested. RPW, moving into one-third of the length of one arm and remaining there for at least 30 s, was classified as “making a choice.” RPW not entering either arm during 5 min was recorded as “no choice.” The data were collected from at least 60 successful “make choice” adults, and the data from “no choice” were excluded and analyzed because we were not sure that RPW was repelled by odor or it did not make a behavioral response at all. Statistical differences were evaluated *via* multiple *t* tests.

## Results

### Characteristics of the Seven ORs in the Rfer-Specific Clade

**FIGURE 1:**
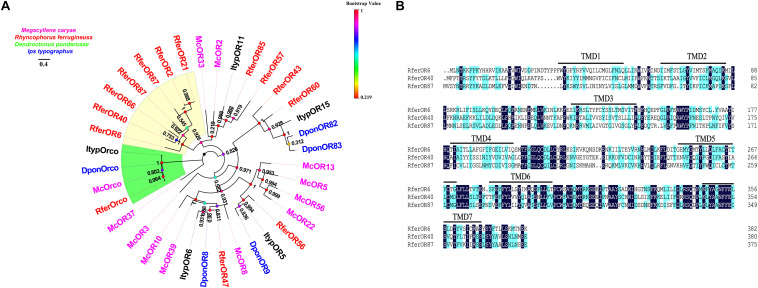
Phylogenetic comparison of odorant receptors (ORs) from four beetle species. **(A)** Maximum likelihood tree of selected OR sequences from *Rhynchophorus ferrugineus* (Rfer, red), *Megacyllene caryae* (Mc, pink), *Ips typographus* (Ityp, dark), and *Dendroctonus ponderosae* (Dpon, blue). Bootstrap based on 1,000 replicates and rooted with the conserved odorant receptor co-receptor (Orco) lineage. *RferOrs* studied in the manuscript are shown in a yellow background. **(B)** Sequences alignment analysis of *RferOr6*, *RferOr40*, and *RferOr87*. The black background indicates the same amino acid sequence of the three genes, and the blue background indicates the same hydrophobicity of the amino acid sequence of the three genes.

We chose the initial candidate *RferOrs* that belong to Group 2 family in beetle species according to previous studies (Antony et al., 2016, 2021; Mitchell et al., 2020). The phylogenetic trees were constructed with four beetle species including *R. ferrugineus* (Rfer, red), *M. caryae* (Mc, pink), *I. typographus* (Ityp, dark), and *D. ponderosae* (Dpon, blue) and analyzed by using the maximum-likelihood method (Figure 1A). Orcos were clustered into one branch, and ORs were separated between four species. Seven *RferOrs* were observed as clustered into one branch and obviously showed species specificity. Three of the seven ORs containing a full-length ORF, namely, *RferOr6*, *RferOr40*, and *RferOr87*, encoded proteins of 382, 380, and 376 amino acids, respectively, and predicted to possess 7 TMDs (Figure 1B). *RferOr2*, *RferOr21*, *RferOr66*, and *RferOr67* were excluded due to encoded proteins of 261, 312, 158, and 238 amino acids, respectively. The sequences of *RferOr*6, *RferOr40*, and *RferOr87* have been deposited in GenBank under the accession numbers MW979236, MW979238, and MW979247, respectively. Three candidate ORs shared 52.37% amino acid identity, while *RferOrco* shared 92.01% identity with McOrco, ItypOrco, and DponOrco.

### Tissue Expression Pattern of *RferOr6, RferOr40*, and *RferOr87* Genes

**FIGURE 2:**
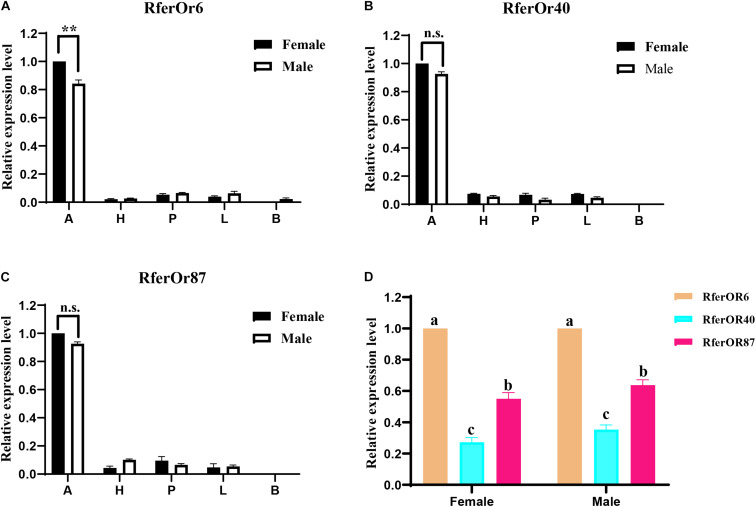
Relative expression levels of three candidate genes in different tissues. **(A)** *RferOr6*. ** indicates the significant difference at the *P* < 0.01 level between sexes (Student’s *t*-test, *n* = 3). **(B)** *RferOr40*. n.s indicates no significant difference between sexes (Student’s *t*-test, *n* = 3). **(C)** *RferOr*87. n.s indicates no significant difference between sexes (Student’s *t*-test, *n* = 3). **(D)** Relative expression levels of antennal expression of *RferOr6*, *RferOr40*, and *RferOr87* (one-way ANOVA). A, antennae; H, heads without antennae and proboscides; P, proboscides; L; legs; B, body. Data represent mean ± SEM.

The RT-qPCR analysis was performed to evaluate the expression levels of three candidate genes in different tissues of male and female RPW (Figure 2). It was observed that *RferOr6*, *RferOr40*, and *RferOr87* were significantly highly expressed in antennae than in other tissues, such as heads, proboscides, legs, and body (Figures 2A–C). *RferOr6* obviously showed higher expression level in female antennae than male antennae (*P* < 0.01), while *RferOr40* and *RferOr87* showed no significant difference between sexes (*P* = 0.57, *P* = 0.25). *RferOr6* was expressed in the male body but not in the female body, while *RferOr40* and *RferOr87* were not expressed in both male and female bodies. According to the relative expression level of three candidate genes in antennae (Figure 2D), *RferOr6* showed the highest expression level, followed by *RferOr87*, and *RferOr40* had the lowest expression level between sexes.

### Functional Characterization of Three Candidate RferOrs in the *Xenopus* Oocyte Expression System

**FIGURE 3:**
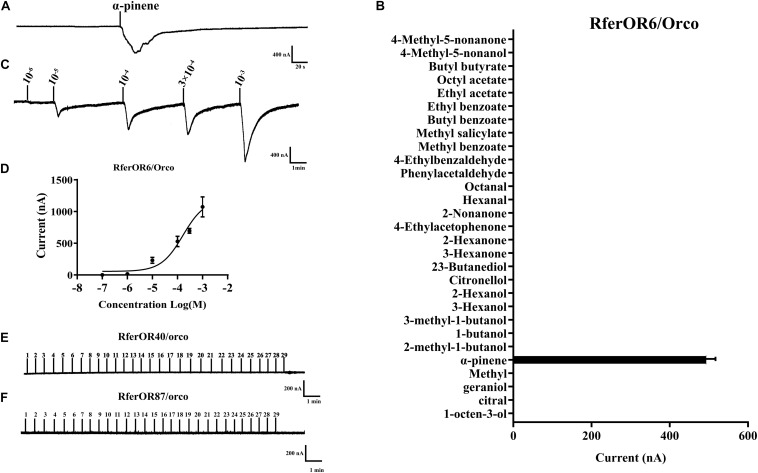
Functional characterization of three candidate genes in *Xenopus* oocytes to stimulation by volatile compounds. **(A)** Inward current responses of *RferOR6*/*RferOrco Xenopus* oocytes in response to 29 tested compounds at the concentration of 10^–4^ M. **(B)** Response profile of *RferOr6*/*RferOrco Xenopus* oocytes. The response value is indicated as means ± SEM (*n* = 6). **(C)** *RferOr6*/*RferOrco Xenopus* oocytes stimulated with a range of α-pinene concentrations. **(D)** Dose–response curve of *RferOr6*/*RferOrco Xenopus* oocytes stimulated by α-pinene. α-pinene EC50 = 9.447 × 10^–5^ M. Error bars indicate SEM (*n* = 6). **(E)** Inward current responses of *RferOr40*/*RferOrco Xenopus* oocytes in response to 29 tested compounds at the concentration of 10^–4^ M. **(F)** Inward current responses of RferOr87/*RferOrco Xenopus* oocytes in response to 29 tested compounds at the concentration of 10^–4^ M.

To characterize the functions of three candidate *RferOrs*, *RferOr6*, *RferOr40*, and *RferOr87* were co-expressed with *RferOrco* in *Xenopus* oocytes and recorded by using the TEVC system. Twenty-nine compounds (Table 1) including non-host plant volatiles, host plant volatiles, and aggregation pheromone compounds were chosen as potential ligands of three candidates of *RferOrs*. The results indicated that *RferOr6*/*RferOrco* was narrowly responded to α-pinene, with responses of about 480 nA (Figures 3A,B). In dose–response studies, we assayed the responses of *RferOr6* to a range of α-pinene concentrations (Figure 3C), and the EC50 value for α-pinene was 9.447 × 10^–5^ (Figure 3D), while no response has been observed in *RferOr40*/*RferOrco* and *RferOr87*/*RferOrco* (Figures 3E,F).

### Behavioral Response to α-Pinene of RPW

**FIGURE 4:**
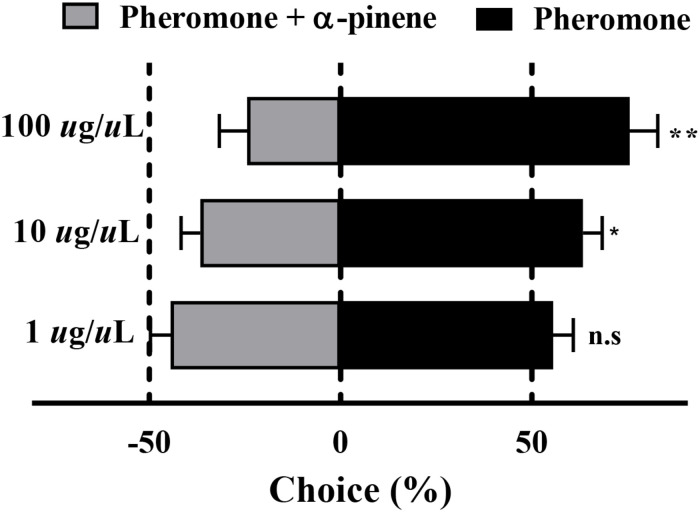
Behavioral response of RPW to α-pinene. The significantly reduced attraction of RPW was elicited by mixed compounds at the concentrations of 10 and 100 μg/μl. No obvious difference was observed at 1 μg/μl. Statistical differences were analyzed by multiple *t* tests. n.s > 0.05, *P < 0.05, and **P < 0.01. Data indicate the mean ± SEM, *N* = 60.

The results of the Y-tube olfactometer assay showed that with the increased concentration of mix G, an obviously repelled behavioral response was observed (Figure 4). There was no preference between the pheromone and mix G at the concentration of 1 μg/μl (*P* = 0.07), while RPW was repelled by mixed compounds at the concentration of 10 or 100 μg/μl (*P* < 0.05). More than 71% of RPW moved toward the side that contained only pheromone at 100 μg/μl and obviously showed a difference with mix G (*P* < 0.01). A similar lower proportion (62%) was observed at the concentration of 10 μg/μl (*P* < 0.05).

## Discussion

Only suitable hosts can obtain sufficient nutrition for growth, development, reproduction, and other series of life activities, while unsuitable hosts can cause insects poisoning and malnutrition (Chapman, 2003). Complex olfactory chemical cues are encoded by a large and divergent repertoire of chemoreceptor proteins, and ORs are considered to play a crucial role in the process of host and non-host plant volatile discrimination (Fleischer et al., 2018). To our knowledge, the functional characterization of ORs is relatively poorly understood in coleopterans. Only several ORs of *M. caryae* (Mitchell et al., 2012), *I. typographus* (Hou et al., 2020; Yuvaraj et al., 2021), and *R. ferrugineus* (Antony et al., 2021) are broadly known for aggregation pheromone receptors of beetles. Besides, two ORs of *I. typographus* specifically responding to host volatiles (Hou et al., 2020) and one OR of *Holotrichia parallela* responding to three volatiles released by the host tree have been reported (Wang et al., 2020). Still, ORs related to the detection of non-host plant volatiles have not been reported. In this study, from seven ORs in the Rfer-specific clade of RPW, we identified that *RferOr6*, *RferOr40*, and *RferOr87* have complete ORF. The tissue expression pattern showed that three *RferOrs* were mainly expressed in antennae and *RferOr6* was highly expressed in female antennae than male antennae. The functional characterization of three candidate *RferOrs* by using the *Xenopus* oocyte expression system showed that *RferOr6* was strongly and narrowly tuned to α-pinene. The behavioral results indicated that 62 and 71% of RPW were attracted to the odor resource without α-pinene at the concentrations of 10 and 100 μg/μl. It means that RPW showed an effective repelled behavioral response to α-pinene at these two concentrations. Meanwhile, previous studies also indicated that feeding and egg oviposition behaviors of RPW can be repelled by α-pinene (Guarino et al., 2013, 2015). Consequently, our results demonstrated that non-host plant volatile α-pinene may be detected by antenna-biased expressed *RferOr6*, thereby possibly regulating RPW to avoid feeding and egg oviposition on unsuitable hosts.

A recent phylogenetic analysis included 1,181 ORs from 10 species representing the four coleopteran suborders, and one species of the sister order *Strepsiptera* showed OR Group 7 subfamily, which is highly expanded in coleopterans (Mitchell et al., 2020). Not only aggregation pheromone receptors of *I. typographus* (Hou et al., 2020; Yuvaraj et al., 2021) and *R. ferrugineus* (Antony et al., 2021) but also ORs of *I. typographus* responding to volatiles released by the host trees or the symbiotic fungi (Hou et al., 2020) were presented within this subfamily. Therefore, the functional characterization of ORs in beetles tends to focus on this clade. However, less attention on the lineage-specific expansions and odor specificity may evolve alone with OR-lineage radiations. According to previous studies, targeted *RferOrs* for following the functional characterization are part of an Rfer-specific OR-lineage of RPW, and this lineage belongs to the monophyletic OR Group 2 subfamily in beetles (Mitchell et al., 2012, 2020; Yuvaraj et al., 2021). The phylogenetic tree shows that Orcos were clustered into one branch, which is highly conserved among different insects. Also, ORs vary significantly between four beetles with low sequence homology (Figure 1A). Seven *RferOrs* were observed as clustered into one branch and obviously showed species specificity and this suggests that these ORs are related to certain life activities of RPW. Interestingly, previous study suggested that two aggregation pheromone receptors (i.e., McOr5 and McOr20) of *M. caryae* are scattered in the Group 2 subfamily (Mitchell et al., 2012). In contrary to our initial prediction, we obtained these receptors possibly related to aggregation pheromone compounds of RPW. In this study, the functional results suggested that *RferOr6* was shown to selectively respond to the non-host compound α-pinene. Our findings indicate that ORs from this clad may detect compounds from various biological sources. Hence, the large-scale functional characterization of the OR repertoires of *R. ferrugineus* and additional species is required to further our understanding of the relationships of OR between species.

Actually, the expression pattern of ORs may vary considerably between sexes, developmental stages, or olfactory tissues, and the expression pattern determines their functions to some extent (Alabi et al., 2014). *RferOr6*, *RferOr40*, and *RferOr87* were highly expressed in antennae and *RferOr6* obviously showed the higher expression level in female antennae than male antennae, while *RferOr40* and *RferOr*87 showed no significant difference (Figure 2), and thus they function in the detection of volatiles. The *Xenopus* oocyte expression system was widely used to study the connection between ORs and volatiles (Fleischer et al., 2018). When three targeted *RferOrs* were co-expressed with *RferOrco*, *RferOr6* had a very high affinity with an EC_50_ of 9.447 × 10^–5^, which indicated that *RferOr6*/*RferOrco* was sensitive to α-pinene. Furthermore, *RferOr6*/*RferOrco* that did not respond to other compounds indicated that *RferOr6*/*RferOrco* had a specific response to α-pinene, whereas no responses have been observed for 29 chemicals from *RferOr40* and *RferOr87*. Therefore, our results demonstrated that *RferOr6* was a functional OR possibly used by RPW to detect compounds in non-palm trees.

In fact, many insects generally show a tendency response to host plant volatiles and non-selectivity or repellency to non-host plant volatiles (Mann et al., 2012; Krause Pham and Ray, 2015). For example, *Cupressus lusitanica* and *Eucalyptus saligna* leaf essential oils are promising insecticides and repellents to be used against insect pests of *Tribolium castaneum*, *Acanthoscelides obtectus*, *Sitotroga cerealella*, and *Sitophilus zeamais* (Bett et al., 2016). Several terpene volatiles [(1R)-(+)-α-pinene, (–)-β-pinene, (R)-(+)-limonene and β-myrcene] released by coniferous trees exhibited significant repellent behavioral responses of Leguminosae pest *Colposcelis signata* (Fan et al., 2020). Previous studies indicated that the behavioral response of insects can be adjusted by detecting non-host-specific compounds (like taxonomically specific compounds) or the different ratios of common plant volatiles in non-host plants with the host plants (Pickett et al., 2012). Given that α-pinene is the compound of coniferous trees with a characteristic odor, several species of conifer-feeding beetles were attracted to the conifer monoterpenes (i.e., α-pinene, β-pinene, myrcene, limonene, camphene, and carene) (Chénier and Philogène, 1989). Considering, α-pinene was not one of the volatile compounds of *Phoenix canariensis* (Vacas et al., 2014), it seems reasonable for α-pinene to exhibit significant repellent to RPW. Since studies on palm volatile profiles are limited to some extent, it is not clear whether α-pinene is a compound of other palm plant volatiles or released only when it is harmed by RPW.

## Conclusion

*RferOr6* is highly expressed in the female antennae and possibly be used by RPW to detect α-pinene to regulate females avoiding feeding and egg oviposition on non-palm trees. 1-Octen-3-ol has been known as a repellent of *Meligethes aeneus* F. (Smart and Blight, 2000) and *Dendroctonus* spp. (Pureswaran and Borden, 2004). Repellent activity of geraniol toward a number of other coleopteran species is also reported (Olivero-Verbel et al., 2010). Unfortunately, the key functional response of ORs to these compounds has not yet been identified. Consequently, it is necessary to characterize more functional ORs related to non-palm plant volatiles and provide a fundamental basis for understanding the RPW olfactory molecular recognition mechanism of host plant volatiles in future studies.

## Data Availability Statement

The datasets presented in this study can be found in online repositories. The names of the repository/repositories and accession number(s) can be found below: https://www.ncbi.nlm.nih.gov/, MW979236; https://www.ncbi.nlm.nih.gov/, MW979238; and https://www.ncbi.nlm.nih.gov/, MW979247.

## Author Contributions

GW and YH designed the experiments and revised the manuscript. TJ, ZX, and QJ performed the experiments. TJ wrote the manuscript and analyzed the data. All authors contributed to the article and approved the submitted version.

## Conflict of Interest

The authors declare that the research was conducted in the absence of any commercial or financial relationships that could be construed as a potential conflict of interest.

## Publisher’s Note

All claims expressed in this article are solely those of the authors and do not necessarily represent those of their affiliated organizations, or those of the publisher, the editors and the reviewers. Any product that may be evaluated in this article, or claim that may be made by its manufacturer, is not guaranteed or endorsed by the publisher.

## Footnotes

1
https://topcons.cbr.su.se
