# Supplementary material for: Non-palm Plant Volatile α-Pinene Is Detected by Antenna-Biased Expressed Odorant Receptor 6 in the Rhynchophorus ferrugineus (Olivier) (Coleoptera: Curculionidae)
Source: Front Physiol. 2021 Aug 9;12:701545. doi: 10.3389/fphys.2021.701545 (PMC8381602; doi:10.3389/fphys.2021.701545)
Supplement: Supplementary file 1 [file Table_1.DOCX]

**Supplementary Tables**

**Table S1 Primers used in this study.**

| **Primer name** | **Sequence (5’-3’)** |
| --- | --- |
| **Primers for gene clone**  RferOrco-F  RferOrco-R  RferOR6-F  RferOR6-R  RferOR40-F  RferOR40-R  RferOR-87-F  RferOR-87-R  **Primers for construction expression vector**  RferOrco-F  RferOrco-R  RferOR6-F  RferOR6-R  RferOR40-F  RferOR40-R  RferOR-87-F  RferOR-87-R  **Primers for qPCR**  RferTubulin-F  RferTubulin-R  Rferβ-actin-F  Rferβ-actin-R  RferOR6-F  RferOR6-R  RferOR40-F  RferOR40-R  RferOR-87-F  RferOR-87-R | ATGAACACATTCAAAGTTGCGG  TTATTTGAGTTGTACCAATACCATGAAG  ATGCTGAATTACAAAAAGTTTTTCAAA  CTATTTTTCATTTGTCATTTTACTCAATAAG  ATGTTTCCTACCTATAGATCATATTTCTATAC  TTATTCTTCCATATTCAGGTTACTCAA  ATGGTTTCCTATAATTATAGACGGTATTAC  CTATTCTTCTTCGGAGTTTAGATTGC  TCAgggcccGCCACCATGAACACATTCAAAGTTGCGG  TCAgcggccgcTTATTTGAGTTGTACCAATACCATGAAG  TCAgggcccGCCACCATGCTGAATTACAAAAAGTTTTTCAAA  TCAgcggccgcCTATTTTTCATTTGTCATTTTACTCAATAAG  TCAgggcccGCCACCATGTTTCCTACCTATAGATCATATTTCTATAC  TCAgcggccgcTTATTCTTCCATATTCAGGTTACTCAA  TCAgggcccGCCACCATGGTTTCCTATAATTATAGACGGTATTAC  TCAgcggccgcCTATTCTTCTTCGGAGTTTAGATTGC  GCTACCTTCATCGGCAACTC  CCTTCGCCAAGTGATATAG  AAAGGTTCCGTTGCCCTGAA  TGGCGTACAAGTCCTTCCTG  TTGCTGTCTTACATTATGGC  CCAATCTGCCGAATAAGC  TGCTCTTATTGTGGTCAGT  GTTGAAAGGATACCATGCTT  TCGGTCATTTGTGTAGCA  TGTTCCTGTTAAGTTTGAGC |

Note: The restriction enzyme sites of primers are underlined. Kozak sequences labelled as lowercase. Vector sequences of pT7Ts designed by In-Fusion Cloning technique are in black box.
